# The PULSAR Specialist Care protocol: a stepped-wedge cluster randomized control trial of a training intervention for community mental health teams in recovery-oriented practice

**DOI:** 10.1186/s12888-017-1321-3

**Published:** 2017-05-08

**Authors:** Frances Shawyer, Joanne C. Enticott, Lisa Brophy, Annie Bruxner, Ellie Fossey, Brett Inder, John Julian, Ritsuko Kakuma, Penelope Weller, Elisabeth Wilson-Evered, Vrinda Edan, Mike Slade, Graham N. Meadows

**Affiliations:** 1 Southern Synergy, Department of Psychiatry, School of Clinical Sciences at Monash Health, Monash University, Dandenong Hospital, 126 - 128 Cleeland St, Dandenong, Victoria 3175 Australia; 2 Royal District Nursing Service Institute, 31 Alma Rd, St Kilda, Victoria Australia; 3 Mind Australia, Heidelberg, VIC Australia; 4 Melbourne School of Population and Global Health, University of Melbourne, Parkville, Victoria 3010 Australia; 5 Department of Occupational Therapy, School of Primary and Allied Health Care, Monash University, Peninsula Campus, Frankston, VIC Australia; 6 Department of Econometrics and Business Statistics, Faculty of Business and Economics, Monash University, Melbourne, Australia; 7 Graduate School of Business and Law, RMIT University, Melbourne, VIC 3001 Australia; 8 College of Business, Victoria University, Melbourne, VIC Australia; 9 Institute of Mental Health, School of Health Sciences, University of Nottingham, Triumph Road, Nottingham, NG7 2TU UK

**Keywords:** Recovery, Recovery-oriented Practice, Specialist Mental Health Services, Mental Health, Co-production, Co-design, Training, Psychiatry, Randomized Controlled Trial (RCT), Complex Intervention

## Abstract

**Background:**

Recovery features strongly in Australian mental health policy; however, evidence is limited for the efficacy of recovery-oriented practice at the service level. This paper describes the Principles Unite Local Services Assisting Recovery (PULSAR) Specialist Care trial protocol for a recovery-oriented practice training intervention delivered to specialist mental health services staff. The primary aim is to evaluate whether adult consumers accessing services where staff have received the intervention report superior recovery outcomes compared to adult consumers accessing services where staff have not yet received the intervention. A qualitative sub-study aims to examine staff and consumer views on implementing recovery-oriented practice. A process evaluation sub-study aims to articulate important explanatory variables affecting the interventions rollout and outcomes.

**Methods:**

The mixed methods design incorporates a two-step stepped-wedge cluster randomized controlled trial (cRCT) examining cross-sectional data from three phases, and nested qualitative and process evaluation sub-studies. Participating specialist mental health care services in Melbourne, Victoria are divided into 14 clusters with half randomly allocated to receive the staff training in year one and half in year two. Research participants are consumers aged 18–75 years who attended the cluster within a previous three-month period either at baseline, 12 (step 1) or 24 months (step 2). In the two nested sub-studies, participation extends to cluster staff. The primary outcome is the Questionnaire about the Process of Recovery collected from 756 consumers (252 each at baseline, step 1, step 2). Secondary and other outcomes measuring well-being, service satisfaction and health economic impact are collected from a subset of 252 consumers (63 at baseline; 126 at step 1; 63 at step 2) via interviews. Interview-based longitudinal data are also collected 12 months apart from 88 consumers with a psychotic disorder diagnosis (44 at baseline, step 1; 44 at step 1, step 2). cRCT data will be analyzed using multilevel mixed-effects modelling to account for clustering and some repeated measures, supplemented by thematic analysis of qualitative interview data. The process evaluation will draw on qualitative, quantitative and documentary data.

**Discussion:**

Findings will provide an evidence-base for the continued transformation of Australian mental health service frameworks toward recovery.

**Trial Registration:**

Australian and New Zealand Clinical Trial Registry: ACTRN12614000957695. Date registered: 8 September 2014.

## Background

Recovery-oriented practice involves facilitating a process of change through which individuals who have been diagnosed with mental illness are supported to reclaim their personal identity, live a self-directed life, and strive to reach their full potential [1, 2]. This can be seen as a paradigm shift in specialist mental health service delivery, from a focus on ameliorating symptoms to an approach that recognises people’s strengths, self-capacity and potential for personal recovery, even in the context of ongoing symptoms or disability [3, 4]. The history of the international recovery movement is longstanding and influenced by the consumer movement as well as emerging evidence that challenges more pessimistic assumptions about recovery from severe and persistent mental illness [5, 6]. Note that in this paper we will use the term consumer to refer to a person with a diagnosis of mental illness or who uses mental health services. The impact of this paradigm shift towards recovery can be identified in mental health policy, practice and law in all Australian states and territories, especially in the last 10 years [7], and is gradually transforming services.

### The development of a recovery orientation in mental health in Victoria

Since the concept of recovery first emerged from the consumer movement in the 1970s and 1980s, the reorientation of mental health policy and services toward recovery has gained increasing momentum in the Victorian mental health sector [8]. At the national level, recovery was first formally endorsed in the 2003–2008 Australian National Mental Health Plan [9]. Subsequent developments in the community-managed health sector accelerated the Australian recovery movement over the following decade, including the establishment of early intervention alternatives to inpatient treatment such as the sub-acute Prevention and Recovery Care (PARC) programs. In 2011, the Victorian Government commissioned a framework document supporting the development of evidence-based recovery-oriented mental health services with an emphasis on facilitating personal recovery and dismantling barriers to full participation in community life for people with experiences of mental illness [8]. This was followed in 2014 by the implementation of a new Mental Health Act in Victoria that established recovery as a fundamental guiding principle in the provision of mental health care. Recovery has thus emerged as a core feature of contemporary reform to mental health service planning and delivery at both the state and national level. Complementing these developments has been an increasing emphasis on the importance of “co-design” or “co-production” to ensure that consumers, families and carers are centrally involved in the design, development and delivery of mental health services [10, 11]. Despite these reforms, the mental health care system still has a long way to go in being responsive to the cultural and linguistic diversity of the Australian population [12]. Little is known about the effectiveness of mental health interventions across people of different cultural and linguistic groups and whether the contemporary emphasis on recovery orientated practice is having the presumed positive impacts on consumers.

### REFOCUS

The value and efficacy of system-wide transformation to focus on recovery is yet to be empirically established in Australia. In the UK, a staff training intervention (called REFOCUS) promoting personal recovery and enabling organizational change in specialist mental health services has been developed and trialled [13]. Based on a systematic review and narrative synthesis of existing literature on recovery, the REFOCUS team developed a conceptual framework of personal recovery, which identified five key recovery processes denoted by the acronym “CHIME”: Connectedness, Hope, Identity, Meaning, and Empowerment [14]. This conceptual framework informed the development of a team-based training intervention for community mental health teams in England that was designed to promote recovery through changes in staff and team skills, knowledge, behaviour, values and relationships with consumers [13]. In a large scale cluster randomized controlled trial (cRCT), the outcomes of usual care plus the REFOCUS intervention were compared with usual care only (control) in 27 community mental health teams delivering services to adult patients with psychotic disorders. The primary outcome, personal recovery as assessed using the Questionnaire about the Process of Recovery (QPR), did not differ between the REFOCUS intervention group and controls, although staff-rated functioning and unmet needs did improve in the intervention group [15]. The authors suggest that implementation was the central challenge, and when high-participating teams were compared with low-participating teams, higher participation was associated with higher staff-reported recovery-promotion behaviour and improved consumer-rated QPR. Challenges in implementing the intervention at the team level included variability in staff participation and adherence to recovery-oriented training procedures [16] along with the diluting effects of staff turnover. Participant attrition was higher than anticipated (26% vs 7%) resulting in a reduction in planned statistical power. A further proposed possible reason for the overall lack of difference between the intervention and control group on recovery outcomes is that the 12-month timeframe may have been of insufficient length for the intervention to take effect. On average, patient participants had been using mental health services for more than 15 years, suggesting the possibility of established staff-consumer relationships and entrenched ways of relating to services and problems that may take longer than 1 year to change [17].

### PULSAR

In the Principles Unite Local Services Assisting Recovery (PULSAR) Specialist Care trial, REFOCUS training materials and the research design have been adapted to enable the testing of the intervention in specialist mental health care services in Australia. The PULSAR staff training intervention aims to train community mental health staff in recovery-oriented practice, so as to embed recovery principles in mental health service delivery in the southern metropolitan region of Victoria, Australia. The PULSAR Specialist Care trial is part of the broader PULSAR research program focused on promoting recovery-oriented practices which also includes the Australian primary care sector [18]. The study components were co-designed with a consumer academic (VE), and the involvement of PULSAR Lived Experience Advisory Panel (LEAP) created for the project (see *Leadership structure* below) and facilitated by VE. This paper outlines the PULSAR Specialist Care study protocol. The protocol follows the Standard Protocol Items: Recommendations for Interventional Trials (SPIRIT) guidelines [19].

### Objectives

Using a mixed methods design, the primary objective of the PULSAR Specialist Care study is to evaluate whether adults accessing study cluster specialist mental health services where staff receive the recovery-oriented practice training intervention report superior recovery outcomes compared to adults accessing services where staff have not received the intervention. The following research questions will be addressed:

1. From pre- to post-intervention, do consumers in intervention clusters report greater improvements in a) personal recovery b) health and well-being, and c) perceived need and satisfaction with services compared with consumers receiving care in control groups?
2. From pre- to post-intervention, do ethnic minority consumers in intervention clusters report greater improvements on measures of personal recovery compared to ethnic minority consumers receiving care during control phases?

A nested qualitative sub-study involving consumers and staff will be conducted. For consumers, the research question that will be addressed is:

1. How do consumers experience and view the support for their recovery in services where the PULSAR training has taken place?

For staff, the research questions to be considered are:

1. What factors help and hinder working in a recovery-oriented manner, from the perspective of staff who have received the PULSAR training intervention?
2. What experiences and dilemmas are encountered when implementing recovery-oriented practices within different parts of the Australia mental health service system, and what strategies are used to address the issues identified?

A nested process evaluation sub-study aims to examine quantitative and qualitative data including documents and processes related to training implementation and the uptake of new ways of working in order to articulate important explanatory variables relating to clusters that affected the rollout of the intervention and potentially influenced the study outcomes.

## Methods

### Overall design

**Fig. 1:**
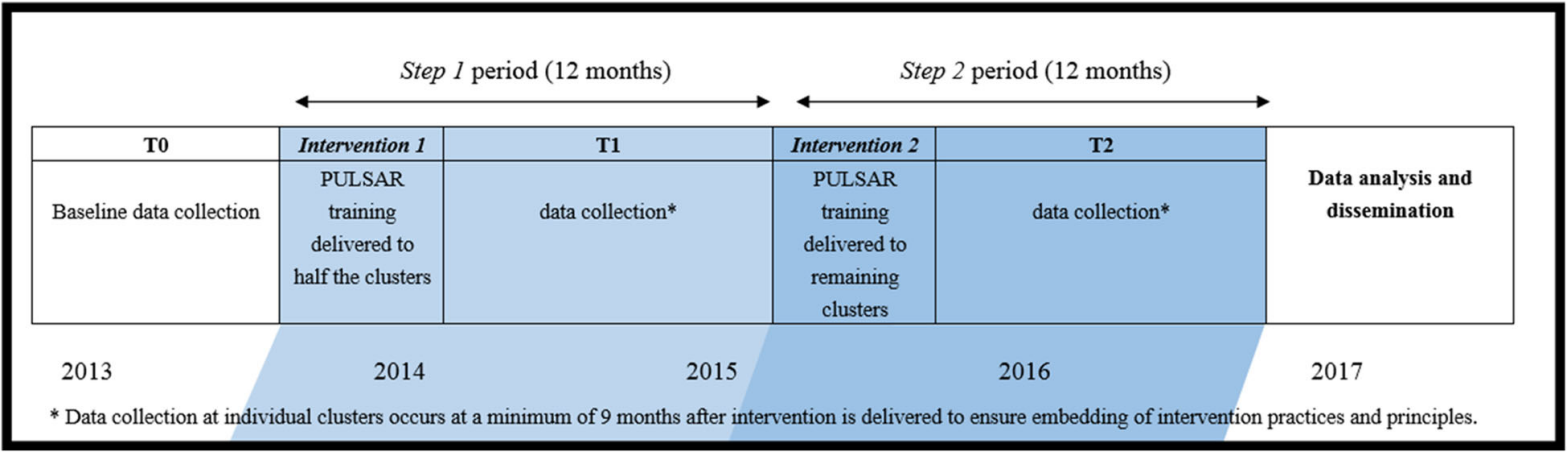
PULSAR Specialist Care study design and planned timeline Notes. Indicated at the *bottom* is the study year. The two-step cluster randomise control trial is shown: half the clusters receive the intervention in step 1 and the remaining clusters receive the intervention in step 2. For a summary of the study evaluations see Table 1

The PULSAR Specialist Care project is one of two multisite two-step stepped-wedge cRCTs within the broader PULSAR research program [18]. The study design of the PULSAR Specialist Care project is a mixed methods design incorporating a two-step stepped-wedge cluster randomized controlled trial (cRCT) examining cross-sectional data from three phases, and nested qualitative and process evaluation sub-studies (see Fig. 1 and Table 1). Co-design with a consumer academic who is an author (VE) was a key design driver, and this co-design began early when the initial protocols were drafted for the application for funding.

**Table 1:** Evaluation plan for the PULSAR Specialist Care study

	Sub-study name	Evaluation design	Unit of analysis	Number	Number at each time point	Number in each cluster at each time point	Detectable differences		
							Primary outcome, QPR	Secondary outcome, WEMWBS	Secondary outcome, INSPIRE
cRCT (quantitative data)	Stream 1 (primary analysis)	cross-sectional cRCT (complete step-wedge)	Consumers (mail-out)	756	252 at baseline 252 at step 1 252 at step 2	18	6.34 (medium effect)	NA	NA
	Stream 2	pre- and post-intervention (incomplete step-wedge)	Consumers (interviews)	252 (stream 1 subset)	63 at baseline 126 at step 1 63 at step 2	9	7.68 (medium effect)	4.80 (medium effect)	7.72 (medium effect)
	Stream 3	longitudinal, (same participant, 12-mths apart pre- and post-intervention)	Consumers with diagnosis of psychosis (interviews)	88 (stream 2 subset)	44 at baseline & step 1 44 at step 1 & step 2	6–7	10.94 (medium-large effect)	6.84 (medium-large effect)	11.28 (medium-large effect)
Nested Qualitative study (qualitative data)			Consumers Staff	20–24 20–24	10–12 at step 1 10–12 at step 2	Nested sub-study examining qualitative data collected in study interviews and focus groups			
Nested Process evaluation (both quantitative & qualitative data)			Consumers & staff	The process evaluation assesses a specific set of qualitative, quantitative and documentary data relating to each cluster.					

Notes. The primary analysis examines the primary outcome– the Questionnaire about the Process of Recovery (QPR) - collected in the two-step stepped-wedge cluster randomized controlled trial (cRCT). A subset of consumers in the cRCT participate in study interviews where secondary outcomes measuring well-being, service satisfaction and health economic impact are collected In a yet another subset in the cRCT, longitudinal data are collected via interviews that are 12 months apart from consumers with a diagnosis of a psychotic disorder. Qualitative and process evaluation sub-studies are nested within the overarching cRCT and include information from consumers and staff.

A cluster randomized design was selected to minimize the threat of contamination between treatment and control groups, as the intervention is administered at the service (cluster) level [15, 20], these clusters being specialist mental health care services in Melbourne, Victoria (see Table 2). The stepped-wedge design was implemented for pragmatic and ethical reasons; in addition to allowing the intervention to be staggered across study clusters, the stepped design offers the ethical advantage of enabling all participating clusters to receive an intervention that is predicted to be beneficial [21]. A mixed method design was utilized for reasons including that the integration of quantitative and qualitative methods provides a richer dataset than can be gained with either approach alone [22, 23]. This in turn allows for a comprehensive and multifaceted evaluation of the project outcomes, as well as further explaining those outcomes and enhancing the project’s credibility. The latter is an important consideration in large-scale projects involving multiple stakeholders, as is the case with the PULSAR project [23]. There are additional benefits to using qualitative and quantitative methods at different stages in the intervention trial; for example, exploratory qualitative techniques were initially used to identify potential obstacles to implementing recovery-oriented practice in service settings and to inform the intervention design, while quantitative methods are used to evaluate the effectiveness of the training intervention. Furthermore, following the intervention trial, qualitative methods are being used to gain a more nuanced understanding of staff experiences of implementing recovery-oriented practices and consumer views of these practices across different settings within Victoria’s mental health service system.

**Table 2:** Cluster sites and stratification factors

Cluster	Team/service	Organisation	Site	Strata	Team/service description
Cluster 1	Crisis assessment & treatment team	Monash Health	1	A	Crisis Assessment and Treatment Teams (CATTs) provide urgent assessment and short-term treatment to people in psychiatric crisis and play a key role in triaging admissions to hospital.
Cluster 2	Crisis assessment & treatment team	Monash Health	2	A	
Cluster 3	Mobile support and treatment service + Community Care Unit	Monash Health	3 4	B	Mobile Support and Treatment Teams (MSTs) provide intensive long-term support through assertive outreach to people with prolonged and severe mental illness with associated high levels of disability. Community Care Units (CCUs) provide medium to long-term residential rehabilitation in a home-like environment.
Cluster 4	Mobile support and treatment service + Community Care Unit	Monash Health	5 6	B	
Cluster 5	Community Mental Health Service	Monash Health	7	C	Community Mental Health Service (CMHS) provide non-urgent assessment, treatment, case management, and continuing care support to people living in the community over varying periods of time.
Cluster 6	Community Mental Health Service	Monash Health	8	C	
Cluster 7	Continuing Care	Monash Health	9	D	Continuing Care Teams (CCTs) provide non-urgent assessment, treatment, case management, and continuing care support to people living in the community over varying periods of time.
Cluster 8	Continuing Care	Monash Health	10	D	
Cluster 9	PARC – Adult + PARC – Extended	Mind Australia	11 12	E	PARC services (including youth, adult and extended PARCs) provide short-term residential support and treatment to assist in averting acute inpatient admission or facilitate earlier discharge.
Cluster 10	PARC – Youth	Mind Australia	13	E	
Cluster 11	PARC Ermha	Ermha	14	F	
Cluster 12	PARC Ermha	Ermha	15	F	
Cluster 13	Community outreach services	Mind Australia	16	G	Community Outreach Services provide a range of individualized psychosocial support and recovery services.
Cluster 14	Community outreach services site 1 Community outreach services site 2	Ermha	17 18	G	

Note. Clusters are stratified by the team/service type and composition: i.e. there are seven different strata

The PULSAR Specialist Care trial will take 4 years to complete, beginning in 2013 and concluding in 2017, see Fig. 1. The original study protocol (documented in the Australian New Zealand Clinical Trials Registry, or ANZCTR) was developed over a period of 18 months in consultation with Chief Investigators (CIs) and an advisory committee comprised of representatives from local specialist and community care organizations, consumers and family/carers, and experts in legal, cultural and educational aspects of mental health service delivery in Victoria. Although minor adaptations have been made to the original protocol, as outlined below, the over-arching two-step stepped-wedge cRCT design remains unchanged and the trial is on target to reach completion within the anticipated 4 year timeframe. All adaptations to the study protocol were considered by, and required the approval of, the appropriate Module Committee governing the relevant aspect of the project (see *Study leadership* section) along with the governing Human Research Ethics Committees.

The PULSAR training intervention is being delivered to 14 specialist mental health care clusters (see Table 2), with clusters randomized to receive the intervention 12 months apart, as shown in Fig. 1. To ensure that cluster types are balanced, stratified randomization was applied, for the strata see Table 2.

### Explanation for choice of comparators

The design was developed to combine the rigor of a cluster randomized trial with the pragmatic approach of the stepped wedge design to implement and evaluate the intervention at all sites [20, 21, 24]. Control sites are those that are yet to receive the intervention. Since all sites eventually receive the intervention, data from sites in control phases will be compared with data from sites that have received the intervention. There are no study restrictions on the care provided in control phases. Treatment as usual is described later under the heading “Control”.

### Study setting and clusters

In Victoria, specialist mental health services include area-based clinical services comprising a range of teams and service types, in particular, inpatient units and community-based continuing care and treatment teams, as well as Mental Health Community Support Services (MHCSS) [25] that provide residential and outreach support. The study setting is the catchment of Monash Health, the largest public health care provider in Victoria, which provides services to a population of over 950,000 in the South-Eastern suburbs of Melbourne, and encompasses a greater population of 1.34 million people [26]. The Monash Health catchment area includes the City of Greater Dandenong, the most culturally diverse municipality in Victoria [27]. Three organisations that operate within the Monash Health catchment are involved in the study including: Monash Health Specialist Clinical Mental Health Services and, from the MHCSS sector, Mind Australia and Ermha.

Fourteen participating specialist mental health care clusters are spread over 18 adult community-based mental health service sites. See Table 2 for a description of study clusters.

### Participants

#### Levels of staff participation

Staff participate in the PULSAR training intervention but no data from individual staff members are collected as part of the cRCT. However, staff are requested to complete a training evaluation at the conclusion of the training and this data will be examined in the process evaluation sub-study. Staff may volunteer for the nested qualitative sub-study.

#### Staff selection criteria

Staff at the study clusters who receive the PULSAR training intervention must fulfil the following inclusion criteria: (a) working on a part-time or full-time basis within the team in a direct service capacity (and not employed on a casual basis); (b) have an active case load with consumers who are recruited into the evaluation. Staff are ineligible if they are also working in a non-intervention cluster at the time of training (to reduce risk of contamination). Similarly, staff are eligible to participate in the nested qualitative sub-study if currently working at services where the training intervention has been provided; and ineligible if they are either not working at a participating cluster site (even if they have completed the training) or no longer work in a direct service role.

#### Levels of consumer participation

In line with the presumption of capacity endorsed by the Victorian *Mental Health Act 2014* and research indicating that participating in research can lead to positive outcomes for people who experience mental health issues [28, 29], the project was designed to provide consumer participants with the opportunity to self-select into multiple levels of involvement. Consumers consenting to participation in the cRCT are offered three levels of involvement, see the three streams outlined in Table 1. In the cRCT stream 1, we collect primary outcome recovery-focused data from consumers recruited cross-sectionally via the mail at baseline (T0), end of year 1 (T1) and end of year 2 (T2), see Fig. 1. In the cRCT stream 2, we additionally collect secondary and other outcome data assessing mental health and wellbeing, service satisfaction, perceived coercion when accessing services, and health economic impact. This information is collected via face-to-face interviews from participants recruited cross-sectionally at pre or post-intervention. Stream 3 consists of a smaller subset of Stream 2 participants who have a clinical diagnosis of psychosis, e.g., schizophrenia, schizoaffective disorder, or bipolar disorder. Participants in this Stream provide longitudinal data via pre- and post-intervention face-to-face interviews. A pool of research assistants trained in study-specific interview procedures and blinded to intervention status, conduct all Stream 2 and 3 interviews. Consumers who volunteer for the nested qualitative sub-study do not need to participate in the cRCT. Consumer data from the cRCT and qualitative study may be used in the nested process evaluation.

#### Consumer selection criteria

Consumers are eligible for participation in any study component if they are receiving care from teams in the participating cluster services. The inclusion criteria for any study involvement are: (a) aged between 18 and 75 years inclusive at time of recruitment; (b) able to provide informed consent; (c) proficient in English; and (d) have accessed a study cluster in the 3 months prior to data collection. An additional inclusion criterion for participation in Stream 3 of the cRCT (see Table 1) is a primary clinical diagnosis of psychosis, e.g., schizophrenia, schizoaffective disorder, or bipolar disorder, recorded in their medical records. The exclusion criteria for all participants are people who are in prison, people unable to give informed consent, and those unable to speak or read English.

### Participant timelines

An overview of the schedule of enrolment, intervention and assessments is shown in Table 3. For staff who participate in the PULSAR training intervention, the overview for the delivery of this training is shown in Fig. 1. In brief, half the study clusters will organise the training to occur with their staff in step 1 and the remaining clusters in step 2. Participating consumers are offered multiple levels of involvement, see above for a summary of the timeline commitments required for the various study involvements (also see Fig. 1 and Table 1). Data collection at individual clusters occurs at a minimum of 9 months after intervention is delivered to ensure embedding of intervention practices and principles.

**Table 3:** Schedule of enrolment, interventions, and assessments

	Time points		
Project events	T0	T1	T2
Specialist staff enrolment			
Eligibility screen	X		
Informed consent	X		
Randomization	X		
Intervention			
Year 1 clusters		X	
Year 2 clusters			X
PALS			
Year 1 clusters		X	X
Year 2 clusters			X
Consumer recruitment			
Eligibility screen	X	X	X
Survey packs to eligible consumers	X	X	X
Informed consent	X	X	X
Consumer (quantitative) assessment			
cRCT - stream 1			
Demographics	X	X	X
QPR	X	X	X
cRCT – streams 2 and 3	X	X	X
WEMWBS	X	X	X
INSPIRE	X	X	X
PNCQ	X	X	X
GAF	X	X	X
SOFAS	X	X	X
CSQ	X	X	X
MASS	X	X	X
Coercion Ladder	X	X	X
Routinely collected data extracted from service medical files		X	X
Staff qualitative sub-study			
Informed consent		X	X
Individual interview		X	
Individual interview/focus group			X
Consumer qualitative sub-study			
Informed consent		X	X
Individual interview		X	
Individual interview/focus group			X
Process evaluation sub-study^a^			
Examination of specific quantitative & qualitative data in study			X
Source key study documentary notes			X
Examine staff training evaluation sheets		X	X

Notes. For a description of the T0, T1 and T2 time points, see Fig. 1. For an expansion of cRCT stream acronyms see Table 4

### Intervention

The intervention is a training program delivered to staff teams in participating clusters over two-day workshops (either team or organisational groups) or equivalent hours. The intervention is based on a package of tools promoting recovery-oriented practice in mental health care that were developed by the REFOCUS team [30, 31] and adapted for the Australian public clinical mental health care setting and the MHCSS Sector [25]. The adaptation of the REFOCUS materials was guided by consultations with: the REFOCUS research team; staff members from participating specialist care organizations in two group sessions based on the Promoting Action of Research Implementation in Health Services (PARIHS) framework [32, 33]; and LEAP. These consultations aimed to: gauge site readiness for recovery-oriented practice; identify potential facilitators and obstacles to implementing recovery-oriented practice in specialist care settings; examine existing organizational activities or service frameworks that could be modified to support the application of the intervention; determine supervisory strategies that would best facilitate staff uptake of the intervention and ensure the materials were inclusive of consumer issues. The adaptation process was overseen by an advisory committee of representatives from key stakeholder groups to ensure that the content and processes of the PULSAR intervention are sensitive to the Victorian mental health care system as well as the local cultural and legal contexts. Drawing on qualitative analysis of the consultation group transcripts and the advisory committee’s expertise, the adaptation process addressed the following issues: ‘How training is delivered’ (e.g. contextualization of training, training over time, follow-up training and practical tools to keep recovery on the everyday landscape), training content related to REFOCUS elements (e.g. language, listening, common understanding of terms, building on staff strengths) and how staff could access a consumer’s choices and preferences differentiating between language and processes, and a concern over the term coaching. Once these adaptations were agreed upon by the advisory committee, the training materials were considered by LEAP and their changes incorporated by the advisory committee.

The intervention focuses on promoting recovery-based practices to staff that are in addition to standard care, and is comprised of two core components: Recovery-Promoting Relationships and Working Practices.

#### Recovery-promoting relationships

According to a recovery-oriented framework, the working relationship between staff and consumers is crucial to the process of recovery. The intervention develops and supports this relationship by: assisting teams to develop a shared understanding of personal recovery; exploring existing values held by individual workers and the team; developing skills in coaching; and raising the expectations held by consumers that their values, strengths and goals will be prioritised in their relationships with staff members.

#### Working practices

The intervention is centered around three main working practices that form the specific behaviours and recovery supports necessary for building positive, recovery-promoting relationships in mental health care: 1) Understanding values, treatment and support preferences; 2) Assessing and working with strengths; and 3) Supporting goal-striving. Staff are trained to ensure that care planning is based on the consumer’s values, preferences, strengths, and personally valued goals.

The intervention is supported by four implementation strategies: 1) Personal recovery training; 2) Coaching and working practice training; 3) Team manager reflection group; and 4) Team reflection sessions, as well as a set of training materials and compatible working tools. The intervention content and implementation strategies are described in detail in the PULSAR training manual, which is available from the corresponding author upon request.

After receiving the intervention, staff are invited to take part in monthly hour long PULSAR Active Learning Sessions (PALS) with an experienced PULSAR facilitator to discuss and reflect upon their experiences of delivering recovery-oriented practice in the service setting. The sessions support the practice-based implementation of the intervention through providing an interactive and collaborative learning environment for staff, and ongoing access to PULSAR trainers and training resources.

#### Intervention modifications

The delivery of the intervention was modified to account for previously unknown restrictions on the ability of services to release staff for two days of training. In response to these restrictions, the first intervention round for clinical services was developed as a two-day session, with the community services training planned as a separate two-day session in the same week. In addition to the consumer trainer being employed by the project, trainers were sourced from clinical services for the clinical sessions and the community sector for the community sessions. This was anticipated as enabling the inclusion of specialist skills and experience in the delivery of training.

Training in the second round was planned to be subject to further modifications based on analyses of evaluations of the first round of training by both participants and trainers.

#### Intervention dosage

Staff movements are tracked at intervention sites every 3 months from the end of training in order to measure the degree of intervention received or “dosage”. Forms are emailed to site managers every 3 months which requests that site managers list any changes in team members who have or have not undergone the intervention training, including changes to work hours and movements within the organization or externally to other organizations. All employed staff of the services are included as of the end of training census date, whether they were trained with PULSAR or not and whether they were on leave or not. This dosage information will be used in the study analyses.

### Control

The control condition is standard treatment, which is defined as follows:

Monash Health: routine care as governed by the policies and procedures applicable to Monash Health, and which are consistent with the National Standards for Mental Health Services 2010 & Directives as issued from time to time by the Chief Psychiatrist of Victoria and concordant with the Mental Health Act 2014.

MHCSS: a non-clinical module of care which already has a number of elements concordant with recovery-oriented practice, and which we will be exploring whether can be further improved by the PULSAR intervention.

### Measures

Both quantitative and qualitative data collection occurs in this study, see Tables 1 and 4. The primary and secondary outcome measures were chosen as they are consumer-rated measures of personal recovery and well-being. Since personal recovery is something experienced rather than assessed by an expert, self-report measures were appropriate for the study end-point.

**Table 4:** Primary, secondary and other outcome measures

Quantitative (Consumer) data collected in the cRCT	
Primary outcome	1. Questionnaire about the Process of Recovery (QPR)
Secondary outcomes	2. INSPIRE questionnaire [13] 3. Warwick-Edinburgh Mental Well-being Scale (WEMWBS) [36]
Other measures	4. Participant Demographic Record 5. Health economic record 6. Days out of role 7. Days absent from work 8. Service utilization questionnaire 9. The Perceived Need for Care Questionnaire (PNCQ) [38] 10. Client Satisfaction Questionnaire [39] 11. Mind Australia Satisfaction Survey [40] 12. The Coercion Ladder [41] 13. Global Assessment of Functioning Scale [42] 14. Social and Occupational Functioning Assessment Scale [42, 43]
15. Routinely collected information in service medical files (data in the year prior to interview):	
	Health of the Nation Outcome Scales (HoNoS; 12 item clinician-rated measure of social disability) [39];
	Basis 32 (consumer-rated);
	LSP16 (clinician-rated); and Focus of Care (clinician-rated).
	Diagnosis information
	Number of community/outpatient mental health contacts: • Care teams involved (discipline) • Location of contact • Date and time of contact • Focus of care for the above
	Number of inpatient mental health admissions: • Inpatient facility type, and Length of Stay (LOS) • Legal status e.g. involuntary admission, etc. Any other relevant mental health related data recorded in electronic file.
Qualitative (Consumers and staff) sub-study data	
Consumer qualitative data	
Individual interviews	
Focus groups	
Staff qualitative data	
Individual interviews	
Focus groups	
Process evaluation sub-study data^a^	

The process sub-study assesses a specific set of study qualitative, quantitative and documentary data relating to each cluster. Includes the data collected from staff after participation in the PULSAR training.

#### Primary outcome measure

The primary outcome measure is the Questionnaire about the Process of Recovery (QPR [34]), a 22-item consumer-rated questionnaire used to assess experience of personal recovery. The QPR comprises of two subscales: Intrapersonal recovery processes (17 items) and Interpersonal recovery processes (5 items), with each item being rated on a 5-point Likert scale ranging from 0 (disagree strongly) to 4 (agree strongly). A higher score indicates increased recovery [34]. The QPR subscales have good internal consistency (Intrapersonal: *r* = 0.94; Interpersonal: *r* = 0.77), test-re-test reliability (Intrapersonal: *r* = 0.874, *p* = 0.001; Interpersonal: *r* = 0.769, *p* = 0.001), and construct validity [34].

#### Secondary outcome measures

There are two secondary consumer-rated outcomes. The 27-item Importance of services in recovery questionnaire (INSPIRE) assesses recovery support from a worker [35]. The two sub-scales of INSPIRE are: Supporting personally defined recovery (Support sub-scale; 20 items) and Working relationships (Relationship sub-scale; 7 items). Items in the Support sub-scale are first rated for whether they are important for the consumer’s recovery (Yes/No). If rated Yes, the item is additionally rated on either a 5-point Likert scale ranging from 0 (Not at all) to 4 (Very much) or as ‘I do not want support from my worker with this’. The Relationship sub-scale is rated on a 5-point Likert scale ranging from 0 (Strongly disagree) to 4 (Strongly agree). The measure is scored by converting the mean of all Likert ratings to a percentage ranging from 0 (low support) to 100 [35].

The Warwick-Edinburgh Mental Well-Being Scale (WEMWBS) is a 14-item scale designed to assess functional and emotional well-being and appraise programs targeted towards the improvement of mental well-being [36]. The scale is rated on a 5-point Likert scale ranging from 1 (None of the time) to 5 (All of the time), providing a total score ranging from 14 to 70. A higher score indicates a higher level of mental well-being.

#### Other measures

Additional measures administered to consumers in streams 2 and 3 of the cRCT include:

- *Participant Demographic Record.* See Table 5 for demographic variables. Response categories for the ethnicity variable were chosen to represent the most common cultural/ethnic groups residing in the Monash Health catchment, sourced from the relevant local government websites. An abbreviated demographic record is included in stream 1 and includes sex, age, country of birth, year of arrival if born overseas, ethnicity, main language, and length of time the consumer has used mental health services at their current service site.
- *Health economic record.* This includes questions about occupation and income.
- *Days out of role.* This item assesses the impact of mental health problems on normal daily activities over the last 30 days.
- *Days absent from work.* This item captures the number of days absent from on usual work or occupation over the last 30 days due to illness or disability, and mental health problems.
- *Service utilisation questionnaire*. This includes questions about service use, including overnight stays in hospital and healthcare consultations, adapted from the 2007 Australian National Survey of Mental Health and Wellbeing [37]. Information about current prescription and non-prescription medication is also collected.
- *The Perceived Need for Care Questionnaire (PNCQ).* This measure classifies the consumers’ perception of their need for care according to four levels: no need, unmet need, partially met need and met need. The PNCQ enables systematic assessment of perceptions of service delivery, especially in relation to mental health service evaluation [38].
- *Client Satisfaction Questionnaire (CSQ).* This consumer-rated measure assesses client satisfaction with the mental health services provided [39].
- *The Mind Australia Satisfaction Survey (MASS).* The MASS is a consumer-rated measure developed by Mind Australia to evaluate overall satisfaction with services provided, individual outcomes associated with service use, and the effectiveness of staff-consumer partnerships in mental health care service delivery [40].
- *The Coercion Ladder*. This visual analogue ladder scale provides a measure of consumers’ perception of coercion in their mental health service interactions including both a hospital and community services version [41].
- *The Global Assessment of Functioning Scale (GAF).* The GAF is a researcher-rated measure of an individual’s level of social, occupational and psychological functioning. The scale ranges from 0 to 100 with a lower score indicating a lower level of functioning [42].
- *The Social and Occupational Functioning Assessment Scale (SOFAS)*. This researcher-rated measure provides an indication of an individual’s level of functioning that is not directly influenced by the severity of a psychological condition and includes impairments caused by both physical and mental health conditions. The scale ranges from 0 to 100 with a lower score indicating a lower level of functioning [42, 43].

**Table 5:** Individual and cluster-level variables available for multivariable analysis

Variable	Description
Individual level	
Demographics	
Sex	Sex of consumer.
Age	Age of consumer at survey completion date.
Country of birth	Country of birth of consumer.
Year of arrival	Year of arrival in Australia if born overseas
Ethnicity	Ethnic or cultural group that the consumer identifies with.
Main language	Main language spoken at home.
Marital Status	Marital status of consumer.
Children	Number and age of any children.
Living situation	Current living situation of consumer.
Education	Education level of the consumer.
Highest qualification	Highest qualification attained by the consumer.
Mental health service use	Length of time consumer has used mental health services.
Health economics	
Employment	Current working status of the consumer.
Income	Usual weekly income of consumer, after tax, from all sources of employment and all sources excluding paid work.
Days out of role	Number of days in the past month that the consumer was totally or partly unable to carry out normal activities because of mental health problems.
Days absent from work	Number of days in the past month that the consumer was absent from work due to illness or disability, and due to mental health problems.
Medication information	Prescription and non-prescription medications taken regularly by the consumer.
Hospitalizations	Number of hospital admissions for physical problems and for mental health problems, including number of nights in total and reasons for most recent admissions.
Consultations with health professionals	Number and length of consultations with health professionals for physical health and mental health problems.
Other	‘Other’ measures that are listed as 9 to 15 in Table 4 above may be investigated as independent variables when relevant. For example, the primary outcome recovery (QPR) scores may be explored for associations with the individual-level scores on the Cohesion Ladder.
Cluster level	
Cluster group	Allocated to receive the intervention at either Step 1 or Step 2.
Intervention status (0/1)	A lag time of 6 months is anticipated until intervention effects are possible. The intervention status variable indicates that this lag time has passed.
Dosage (%)	Intervention dosage.
Time since intervention	All data are time-stamped in relation to the time the intervention was received at the cluster. Time value of “0” is given for the plus/minus 3 months from date of training; “1” for 4-to-6 months post training; “2” for 7-to-9 months post training, etc. Time value of “-1” for 4-to-6 months before training; “-2” for 7-to-9 months before training, etc.
Time	Study month that survey was completed: “1” = month 1, “2” = month 2, etc.
Cluster types - stratification variables, see Table 2	
Crisis assessment & treatment team	
Mobile support and treatment service or Community Care Unit	
Community Mental Health Service	
Continuing Care team	
PARC residential facility (Mind Australia)	
PARC residential facility (Ermha)	
Community outreach service	

Consumers participating in stream 3 who report having no contact with their mental health service in the previous 12 months do not complete measures pertaining to service evaluation (CSQ, MASS, Coercion Ladder: community services version, INSPIRE).

#### Routinely collected information in service files

For participants in streams 2 and 3 of the cRCT, data will also be extracted from routinely collected medical records maintained by participating organisations. Data will be extracted for the 12 months prior to participation in stream 2 or 3. The inclusion of routinely collected data is intended to minimize the burden on participants by reducing the amount of measures that are administered in face-to-face interviews and to enable a detailed understanding of health service and medication use over time.

For Monash Health mental health consumers, this information will be obtained from the organization’s Health Information Services scanned medical records, and will include: information about diagnosis and mental health status (such as ratings on any clinician measures); occasions of contact with services; occasions spent in residential facilities operated by the service; Health of the Nation Outcome Scales (HoNoS; 12 item clinician-rated measure of social disability) [44]; Basis 32 (consumer-rated); LSP16 (clinician-rated); and Focus of Care (clinician-rated), see Table 4.

Some of the above mentioned data routinely recorded in files of Monash Health consumers, for example the HoNoS, are not available in files of consumers from Mind Australia and Ermha. Therefore in these files we will extract service activity information collected from the respective clinical databases including information about diagnosis and mental health status (ratings on any clinician measures); occasions of contact with services; and occasions spent in residential facilities operated by the service.

Diagnosis information extracted from medical files will be used to identify participants who will be invited into stream 3 of the cRCT. Stream 3 participants must have a diagnosis of psychosis, e.g., schizophrenia, schizoaffective disorder or bipolar disorder, see Table 1.

### Sample size

The primary analysis examines QPR data from consumers in the cRCT (stream 1) and requires a total sample size of 756 consumers from 14 clusters over 3 years (see Table 1). This will be sufficient to detect a medium effect size representing a change in QPR score by 6.34, see Table 1. Secondary analyses that examine data from a subset of stream 1 consumers who participate in stream 2 of the cRCT, requires a total sample size of 252 consumers over the study period. This will be sufficient to detect medium effect sizes in the QPR and two secondary outcome measures (WEMWBS and INSPIRE), see Table 1. Additional secondary analyses to examine longitudinal data from a subset of stream 2 consumers who participate in stream 3 of the cRCT, requires a total sample size of 88 consumers over the study period. This will be sufficient to detect medium-large effect sizes in the QPR and WEMWBS and INSPIRE, see Table 1.

Sample size calculations were based on 14 clusters; intracluster correlation coefficient (ICC) of 0.05; significance level set at 0.05; power of 0.80; and available published [34, 36, 45] and unpublished (INSPIRE) data about distribution properties. All sample size calculations indicate the minimum number of participants we aim to recruit and were done using Stata statistical software *stepped-wedge* [46] Version 11, StataCorp. 2009.

### Recruitment

#### Specialist Care Service recruitment

Specialist care services were identified by the clinical and CMHS service partners in the study. A role for the Steering group was to enable initial identification and engagement of services, followed by meetings with chief executive officers or senior managers to discuss the PULSAR study and the possibility of involvement. No services declined to participate.

#### Consumer recruitment

##### Original recruitment protocol

The initial consumer recruitment strategy required local coordinators at each study site to identify potential participants from service administrative and clinical databases using a systematic quota sampling template provided by the study statistician. This method of identifying potentially eligible participants was developed to ensure consumer confidentiality. The site coordinator was then responsible for overseeing the mailing of survey packs to eligible consumers, which contained a 10-page participant information sheet and consent form (PICF), a 2-page questionnaire comprising the QPR and a simple demographic survey (Stream 1 survey), and two color-coded reply paid envelopes. Participants were instructed to return the signed consent form and questionnaire separately in their respective color-coded reply paid envelopes. This strategy was designed to protect participant confidentiality by ensuring that participant data was returned independently of identifying contact information. A unique matching code was printed on each of the forms to allow subsequent data linkage.

The original PICF invited participants to consent to one of four levels of involvement in the study and sign and return the form accordingly. Consent levels were as follows:

- Level 1 consent refers to a participant consenting to the inclusion of a returned Stream 1 survey into the project.
- Level 2 consent refers to a participant providing additional permission for the researchers to access and use relevant routinely collected clinical data.
- Level 3 consent refers to a participant being willing to be contacted for a maximum of two project interviews.
- Future research consent refers to a participant being willing to be contacted to participate in future research.

However, of the 713 letters mailed out using this initial strategy, only 21 letters (2.9%) were returned over the subsequent 5 weeks.

##### Modified recruitment protocol

Due to this low response rate, the consumer recruitment protocol went through a series of adaptations to facilitate greater engagement and flexibility of recruitment strategies. The primary mode of recruitment through mail out was modified to a) allow mail outs of letters of invitation to complete and return the Stream 1 survey form to all eligible consumers of the participating services from each cluster site; b) replace the 10-page PICF in the survey pack with a simple one-page consent to be contacted for a face-to-face interview form, thus requiring implied consent only for return of the mailed questionnaire and demographic form; and c) provide a $10 shopping voucher for all returned questionnaires where contact details are provided.

A range of secondary recruitment strategies to promote consumer response to the mail outs were added and flexibly employed according to the needs of sites. Strategies include, for example, having researchers, including consumer researchers, present at sites to speak about PULSAR; the use of publicity materials such as advertisements, posters or PULSAR-branded materials; and direct contact with clinicians and consumers at participating sites. Considerable care was taken to ensure, as far as reasonably possible, that recruitment strategies were consistent across time points at participating clusters.

### Allocation

#### Sequence generation

Clusters were randomized to receive the intervention at either step 1 or step 2, see Fig. 1. We used stratified randomization to ensure that cluster types were balanced across arms, see Table 2. The method of sequence generation was by simple randomization using an online Research Randomizer for random number generation. Seven randomization keys were created that corresponded to the seven strata. The randomization was performed offsite by an independent researcher during the third quarter of 2014. Investigators, site coordinators, participants and all others are unable to change the randomization key and intervention allocation given to a site.

### Blinding and procedures to minimize bias

As the intervention involves training, specialist mental health care staff are aware of the intervention condition they are allocated to. Efforts are made to maintain the blindness of research assistants for the onsite recruitment and yearly face-to-face assessments for consumers by withholding information about the allocation of training to clusters and by rotating interviewers across interview and onsite recruitment clusters from T0 and T1. After conducting interviews in streams 2 and 3 of the cRCT, research assistants are asked to classify consumer participants into an intervention condition (PULSAR training provided at their site of service in year 1 or year 2) together with any specific reasons for their response and an estimate of their level of confidence in their judgement to assess whether blindness is preserved.

Procedures adopted to minimize other sources of bias include:

- Allocation status is recorded in a separate (linked) database from the database containing process and outcome data;
- Consumer participants are not informed if cluster staff at the service they attend have received the intervention training;
- The stepped-wedge design can reduce contamination of control clusters as staff in all sites know they will eventually receive the intervention [20, 21];
- In recruitment, considerable efforts are made to minimize possible sampling bias by ensuring that all eligible consumers are given the opportunity to participate. For example, the multiple levels of involvement (see above *Levels of Consumer Participation*) are designed to offer maximum flexibility for consumers to participate based on possible fluctuations in mental health; and
- Randomization was performed offsite by an independent statistician according to the procedures described above.

### Data collection

The broad data collection periods are in Fig. 1. As indicated in Tables 1, 3 and 4 and discussed earlier, consumers are offered multiple levels of involvement and can contribute both quantitative data in the cRCT and/or qualitative data in the nested qualitative sub-study. Data collection from staff occurs in the nested qualitative sub-study. The process evaluation sub-study assesses a specific set of existing qualitative, quantitative and documentary data.

### cRCT data collection

In stream 1 of the cRCT, cross-sectional data are collected from mail-outs to consumers at three time points, see Table 1. Stream 1 participants can return a completed QPR/demographic survey anonymously in a provided reply-paid envelope addressed to the researchers if they wish. However, they are invited to provide their contact details on a separate one-page “Participant Contact and Consent Form” if they would like to be mailed a $10 shopping voucher. Participants can additionally indicate on the Participant Contact and Consent Form if they are willing to volunteer for other parts of the PULSAR project, such as a face-to-face interview, by signing a “Consent to Future Contact” section.

In streams 2 and 3 of the cRCT, data are collected in structured face-to-face interviews from a subset of stream 1 consumers who consent to future contact, see Table 1 and earlier section *Levels of consumer participation*. A pool of around 12–14 casual research assistants conduct the face-to-face interviews. Prior to conducting interviews, all research assistants attend a compulsory 2 day training workshop facilitated by senior PULSAR researchers. This training is conducted annually prior to commencement of fieldwork each year to train new staff and maintain the skills of continuing research assistants. Training modules include: research interviewing skills; research interviewing from the consumer perspective; risk assessment, including consumer safety, risk management, and distress management; staff safety, including aggression and risk management; communication skills; research ethics; home visit protocols; and blindness. The first two interviews with consumer participants are supervised by a senior PULSAR researcher, and research assistants are provided with verbal and written feedback at the end of each interview.

Participants who complete a face-to-face interview are required to provide full written informed consent for both the interview and to the researchers accessing routinely collected data using a revised PICF. Study interviews take around 60–90 min. At the end of T0 and T1 interviews, participants are asked whether they would be interested in completing a follow-up interview approximately 12 months later (for stream 3). If participants are willing to be contacted regarding the follow-up interview, they are asked to provide their contact details, give an indication of whether they are likely to relocate in the coming year, and provide the contact details of any friend or family member who might be able to pass on letters from the PULSAR project should they no longer be contactable. If participants are not available for a follow-up interview after re-contacting attempts have been made by the researchers, no additional data will be collected. Given that participants are the recipients of the intervention through services provided by trained specialist mental health care staff, no protocol for discontinued consumer participants is necessary.

All data are recorded on paper forms which are securely stored at the PULSAR administrative site. Procedures to ensure accuracy of data extraction include double entry from selected hard copy forms, range checks and examination of outliers.

For participants who provide Level 2 consent initially, then later all participants who complete a face-to-face interview, routinely collected medical data is extracted from organization-specific medical records, see Table 4. All identifiers are removed from the service record data and replaced with a code, enabling re-identification for the purpose of linkage with the participant’s interview data.

The privacy of all participants is safeguarded in accordance with the National Statement on Ethical Conduct in Human Research [47]. All data is stored on password protected computer systems located within the secure PULSAR administration site. The study data will be stored for a minimum of 7 years, after which time it may be destroyed. Re-identification codes are only accessible to the core research team responsible for data management. It is possible that participant data may be used in a non-identifiable format in future research.

### Qualitative sub-study data collection

The nested qualitative sub-study investigates mental health staff experiences of implementing recovery-oriented practices following the PULSAR intervention and the challenges involved within Australian mental health settings; it also seeks to explore consumer views of how their recovery has been supported in services where this staff training intervention has taken place. Two semi-structured interview guides for use in staff interviews and consumer interviews were developed, informed by literature on recovery-oriented practice, consumer and service provider expertise within the PULSAR Qualitative Research Steering Group and consultations with PULSAR’s LEAP. These interview guides are used to conduct face-to-face or telephone interviews with mental health staff three to 4 months following the PULSAR training, and with consumers five to 6 months following the PULSAR training. Staff interviews occur prior to consumer interviews on the basis that staff are likely to be aware of their own efforts to implement changes to practice before these become as evident to consumers. Interviews with staff initially explore their understanding of recovery-oriented practice and experiences and challenges encountered in implementing a recovery-oriented framework at a service level. Subsequent interviews will invite participating staff to reflect on the de-identified interview themes, and on facilitators and barriers to implementing recovery-oriented practice in their service settings in an interview or focus group discussion. Similarly, initial face-to-face or telephone consumer interviews focus on their views and experiences of recovery-oriented practice in services where mental health staff have received training, with subsequent interviews inviting consumers to reflect on the de-identified interview themes and on supports for their recovery within and beyond services.

Sample size for the qualitative sub-study is determined sequentially by qualitative sampling processes to ensure diverse perspectives are sought, and to maximize the richness of data obtained, for which we anticipate at least 20–24 staff participants and 20–24 consumer participants will be recruited and interviewed from across the specialist mental health care sites. Recruitment strategies rely on staff and consumers opting into the study based on a convenience sampling approach, informed by the current profile of consumers and staff at participating sites. Efforts are made through the use of varied recruitment strategies including flyers and onsite visits by the researchers to ensure that a diverse range of participants are represented in the evaluation. Following the PULSAR intervention in year 2, the selection of sites, specialist mental health staff and consumers to participate in the qualitative sub-study will be guided by the extent and depth of data gathered in the first year (e.g., whether some service types are under-represented; whether consumers on Community Treatment Orders or staff working with these service users have been recruited).

All qualitative data are audio-recorded (subject to participant consent) or documented in handwritten notes, then transcribed for coding and analysis. Coding will employ both inductive reasoning and an explicit theoretical lens [48]. Thus, qualitative data will be coded and analysed, using a constant comparative method, to identify thematic similarities and differences in participants’ views within and across participant groups. Further, given the PULSAR intervention is informed by CHIME and the REFOCUS recovery-promoting practices (14,29), this theoretical framework will also be used for coding so as to identify how these concepts and practices are spoken about and understood by participants. All transcribed data are de-identified and along with all other PULSAR data are stored in password-protected files within the restricted access electronic files of the PULSAR site.

#### Process evaluation data collection

Given that the recovery-oriented practice involves facilitating a process of change, a process evaluation is crucial to offer explanatory variables that may influence the outcome measures [49]. The nested process evaluation will use quantitative and qualitative data to identify contextual and organisational factors that influence the effectiveness of the intervention.

The process evaluation will provide additional data relating to clusters, drawing on the PARIHS framework dimensions of evidence, context and facilitation [32]. These data can be examined further in regression and other analyses to estimate the extent to which dimensions of readiness for, exposure to, and engagement in the PULSAR program are associated with differentials in outcome measures. The process evaluation study design adopts the recommendations of Moore et al. [50] and acts on the advice of Bhanbhro and colleagues [51] in ensuring our approach is informed by theory and evidence. The interventions in PULSAR seek to change services’ orientation to recovery-oriented practice through influencing the behaviour of clinical staff and adapting the systems in which they work. As Chen and Rossi [52] suggest, we use a theory-driven evaluation approach which is not dependent on a single outcome measure to confirm or refute the effectiveness of the intervention.

Following the guidelines provided by Moore et al. [50], the process evaluation will focus on collecting data that has the potential to surface explanatory variables in the complex path between intervention and outcomes. The theoretically grounded research questions include:

1. What is the role of contextual factors (leadership/support for innovation/readiness for change/organisational support for change/commitment to change and perceived supervisor support for recovery orientation practice) on the adoption of the training and patient outcomes?
2. How does dosage (number of people trained and still working at the facility/number of people attending PALS and still practicing) affect attitude to and uptake of recovery-oriented practice?
3. What is the role of clinical context (Primary or Secondary Care/Community or Acute) on attitudes to and adoption of the intervention and client outcomes?
4. What is the relationship between pre-existing engagement in recovery-oriented practice, on attitudes to training, evaluation of training and transfer of training?

### Statistical analysis

#### Main analysis plan for cRCT

The primary analysis involves evaluating the PULSAR training intervention at the consumer level by examining the QPR data from consumers, see Table 1. The planned data collection schedule has three main periods called T0, T1 and T2, see Fig. 1. Baseline (T0) data collection occurs in the year prior to and 3 months after the step 1 intervention is delivered. The first 3 months after intervention delivery is a period still considered relevant for baseline data collection based on the Kirkpatrick training evaluation model [53] which considers that the embedding of practice change requires a minimum of 9 months after intervention is delivered, including 3 months for consolidation and 6 months for implementation. In the next period called step 1, (T1), data collection occurs during the following 12 months. Then in the next period called step 2, (T2), data collection occurs during the following 12 months. During both T1 and T2 periods, data collection at individual clusters occurs at a minimum of 9 months after the intervention was delivered to ensure embedding of intervention practices and principles, see Fig. 1.

Descriptive statistics will be used to summarize the characteristics of the clusters at baseline and consumer-level variables at time of data collection (see Table 5). Cluster-level variables are those used in the stratified randomization, which are seven types of organizational variations (see Table 2), plus the intervention status of the cluster and the time since (or before) the start of the intervention. The ICC will be calculated and reported.

The analysis of data in a stepped-wedge cRCT is most suitably analysed in mixed-effects models [54]. The primary analysis examines the effect of PULSAR on the primary outcome (consumer-level QPR scores) using a linear mixed-effects model state ‘on an intention-to-treat basis’. The model will include intervention status and time as fixed effects and clusters and consumers as random effects. Normally step one is just to examine intervention – control group differences controlling for cluster, before including covariates. An a priori model-fitting analysis strategy will involve both univariate and multivariable models to be developed based on baseline consumer and cluster-level variables considered statistically significant (*p* < 0.10) or clinically important (e.g., age, sex), see Table 5, and included in the model as fixed. Model fit will be examined by comparing AIC values.

Secondary analyses will examine the effect of PULSAR on secondary outcomes (WEMWBS and INSPIRE) using a linear mixed-effects models to compare the intervention and control periods (pre-intervention).

Estimated intervention effects will be reported as the mean outcome difference for continuous outcomes and Odds Ratio for binary outcomes between intervention and control periods. This can be described as a meta-analysis approach as (in the case of continuous data) the mean change in each cluster will be standardized by using the variance of the outcome measure within that cluster. The estimated intervention effects will be reported with 95% Confidence Intervals and *p* values. Analysis will be conducted using Stata V.14, StataCorp. Stata Statistical Software: Release 14. College Station, TX: StataCorp LP, 2015.

#### Sensitivity analyses

A missing data analysis will investigate any patterns of missingness. For each primary and secondary outcome component with missing data, multiple imputation using multivariate regression with factors of age, gender, time, and intervention status will produce 100 estimates. Sensitivity analyses will be performed using this multiple imputation to account for missing data and then re-running the analyses. Sensitivity analyses will also include the intervention dosage variable described earlier.

#### Economic evaluation

Overall, costs associated with each participant will follow well established health economic principles [53], and cover direct medical costs of illness, plus the labour market effects of illness. Direct medical costs are to be calculated for prescription and other medically recommended non-prescription medications, and hospital and health service contacts. Labour market productivity losses will be imputed using the human capital approach by multiplying reported days off work due to mental illness with an individual’s estimated salary using instrumentation devised by this team for a previous health economic evaluation [54]. Using only days off work due to illness to capture labour market costs captures an important aspect of the cost of illness; however, it is noted that the estimates obtained will be conservative and the true cost will be higher than what we obtain because of other effects of illness such as higher rates of non-participation in employment, or underemployment.

### Leadership structure

The PULSAR project adopts a module based advisory structure, overseen by a project steering group chaired by Principal Investigator (PI), Professor Graham Meadows. Four modules guide and monitor the implementation and evaluation of the project which are chaired by different members of the senior research team. The modules include Adaptation, Implementation, Research and Dissemination.

Based on the REFOCUS project [55, 56], and consistent with the commitment to co-design [10, 11], PULSAR is also supported by LEAP, an advisory group comprising people with either lived experience of mental illness or with experience of caring for someone with mental illness. LEAP was established at the commencement of the PULSAR project and continues to meet during the trial. It provides consumer and family/carer perspectives on the project and ongoing feedback and advice on the trial.

### Specification of safety parameters

No plans were made for a premature stopping of the trial. Apart from any possible breaches to consumer confidentiality, which are classified as moderate risk, all risks to the safety of consumer and specialist staff are classified as minimal.

### Safety oversight

Comprehensive project protocols have been developed to address staff safety, the management of participant distress, suicidal ideation or intent, threat to harm others, and disclosure of previously undisclosed criminal acts. These protocols are readily accessible to all PULSAR research and administrative staff and are reviewed and updated on a continuing basis throughout the trial.

### Dissemination policy

#### Overview

PULSAR takes a multi-tiered approach to dissemination to maximize the translation of knowledge into practice. Dissemination avenues will include: publication of a training manual and associated resources; development of online resources to disseminate project materials to interested parties locally and abroad; publication of project protocols and findings from each component of the project in peer-reviewed literature; production of a regular newsletter updating stakeholders on project progress and outcomes; presentations and national and international conferences; local distribution through partner organizations in the community mental health sector in Victoria; and direct communication of project outcomes to key policy makers.

#### Rights

In relation to copyright issues in dissemination of findings, PI Meadows and CI Slade have agreed to highly accessible publication to maximize dissemination. Specifically, there is no plan to commercialize outputs of this work and so put barriers in the way of use by others. It has been the practice of the multiple research teams involved in the PULSAR proposal to actively seek to make materials widely available as far as possible without cost, and to place barriers in the way of others commercializing such work. For example, the London REFOCUS team have disseminated the REFOCUS intervention in free-to-access booklets and through open access journal articles. The dissemination plan will make the findings widely and readily available along with source training materials.

## Discussion

The PULSAR Specialist Care trial will examine the efficacy of a recovery-oriented practice training intervention for specialist mental health care staff using a two-step stepped-wedge cRCT design. This design is often favoured for such community-based pragmatic trials, as the intervention will eventually be delivered to all participating clusters but can be implemented in stages to manage the practical constraints associated with delivering a large-scale intervention across multiple sites [20, 21]. The challenges encountered in the trial are providing valuable insights on how to facilitate staff adherence to the training and hence the embedding of the intervention into participating services, as well as effective methods for engaging and retaining the participation of consumers. A significant contribution of the work will be the production and dissemination of a package of professional training resources to support the implementation of recovery-oriented practice into community-based mental health services. Although the PULSAR materials have been developed according to the needs of the Australian mental health system and the local social, legal and cultural contexts, we anticipate that these resources will be adaptable to other settings and jurisdictions. In line with the approach taken by our UK partner, the PULSAR materials will be made widely and readily available.

With the current emphasis in mental health policy on refocusing services towards recovery, the results of this trial, including an assessment of clinical, organizational and health economic outcomes, will contribute to the small but growing evidence-base promoting the development of recovery-oriented service frameworks. If successful, it will be the most definitive trial to date in Australia demonstrating that the concept of recovery, and interventions designed to foster recovery-oriented staff behaviour and relationships with consumers, can be operationalized and comprehensively evaluated. Findings, and other information gathered and lessons learned during the trial, will support the continued transformation of the mental health sector towards recovery, ultimately leading to improved outcomes for people with serious mental illness.

## Abbreviations

ANZCTR: Australian New Zealand Clinical Trials Registry
CATT: Crisis assessment and treatment team
CCT: Continuing care team
CCU: Community care unit
CI: Chief investigator
cRCT: Cluster randomized control trial
CSQ: Client satisfaction questionnaire
GAF: Global assessment of functioning scale
HoNoS: Health of the nation outcome scales
HREC: Human Research Ethics Committee
ICC: Intraclass correlation coefficient
LEAP: Lived experience advisory panel
MASS: Mind Australia Satisfaction Survey
MHCSS: Mental health community support services
MST: Mobile support and treatment team
PALS: PULSAR active learning sessions
PARC: Prevention and recovery in community
PARIHS: Promoting action of research implementation in health services
PI: Principal investigator
PICF: Participant information and consent form
PNCQ: Perceived need for care questionnaire
PULSAR: Principles unite local services assisting recovery
QPR: Questionnaire about the process of recovery
SOFAS: Social and occupational functioning assessment scale
SPIRIT: Standard protocol items: recommendations for interventional trials
WEMWBS: Warwick-Edinburgh mental well-being scale
